# Palliative care in undergraduate medical education – consolidation of the learning contents of palliative care in the final academic year

**DOI:** 10.3205/zma001499

**Published:** 2021-09-15

**Authors:** Christina Gerlach, Sandra Stephanie Mai, Irene Schmidtmann, Martin Weber

**Affiliations:** 1 Johannes Gutenberg University Mainz, University Medical Center, III. Med. Clinic & Polyclinic, Hematology, Oncology und Pneumology, Interdisciplinary Department of Palliative Care, Mainz, Germany; 2 Heidelberg University Hospital, Department of Palliative Care, Heidelberg, Germany; 3 Johannes Gutenberg University Mainz, University Medical Center, Institute for Medical Biometry, Epidemiology and Informatics, Mainz, Germany

**Keywords:** undergraduate medical education, palliative care, curriculum, educational measurement, Germany

## Abstract

**Background: **Demographic change and the medical imperative to accompany patients at all times and also in the case of illness leading to death require good basic knowledge of palliative care in large parts of the medical profession. Palliative care has been introduced into undergraduate medical education as a compulsory subject: “interdisciplinary subject 13 palliative care” (Q13). While course concepts for Q13 have already been positively evaluated, assessment of the consolidation and practical relevance of the knowledge taught is lacking.

**Methods: **Assessment of the consolidation of the learning content from Q13 after the practical year (the “practical year” is the sixth and final year of undergraduate medical education in Germany) by means of a survey with a proven questionnaire and integrated qualitative free-text analysis of a cohort of medical students (n=176) who had already participated in an evaluation before and after Q13.

**Results: **The response rate was 96% after Q13 and 45% after the practical year (PY). Teaching was predominantly perceived as more helpful than the PY (p<0.001). Compared to the status after Q13, students rated themselves as less competent after the PY in all areas surveyed, including drug-based pain therapy (p=0.0386). The certainty in informing patients about the incurability of the disease also decreased significantly after the PY (p=0.0117), although the preparation in Q13 was positively highlighted.

**Conclusion: **The knowledge acquired in Q13 could not be anchored in the PY. On the contrary, after initial practical experiences, the students found it challenging to conduct conversations in cases of serious illness and to deal with their own uncertainty in the care of seriously ill patients. Structural factors regarding palliative care in the PY, as well as intrinsic motives of students, such as prior knowledge or motivation to learn, should be analyzed to identify ways to close the gap between theoretical and practical training in general palliative care. To generate samples that are sufficiently representative, future studies on teaching should be easily accessible to students and consider attractive forms of evaluation including electronic methods and social media.

## Zusammenfassung

**Hintergrund: **Der demographische Wandel und der ärztliche Anspruch Patientinnen und Patienten stets und auch bei zum Tode führenden Erkrankungen zu begleiten, erfordern gute palliativmedizinische Basiskenntnisse in weiten Teilen der Ärzteschaft. Mit Wirkung zum Sommersemester 2013 ist Palliativmedizin als „Querschnittbereich 13 (Q13)“ als Pflichtfach in das Medizinstudium eingeführt worden. Während Kurskonzepte zum Q13 bereits positiv evaluiert wurden, fehlt die Beurteilung der Konsolidierung und Praxisrelevanz der vermittelten Kenntnisse.

**Methode: **Beurteilung der Konsolidierung der Lerninhalte aus Q13 nach dem Praktischen Jahr (PJ) mittels Befragung mit einem erprobten Fragebogen und integrierter qualitativer Freitextanalyse einer Kohorte von Medizinstudierenden (n=176), die bereits an einer Evaluation vor und nach Q13 teilgenommen hatten.

**Ergebnis: **Die Rücklaufquote betrug 96% nach Q13 und 45% nach dem PJ. Der Unterricht wurde überwiegend als hilfreicher empfunden als das PJ (p<0.001). Im Vergleich zum Stand nach Q13 schätzten sich die Studierenden nach dem PJ in allen abgefragten Bereichen als weniger kompetent ein, einschließlich der medikamentösen Schmerztherapie (p=0.0386). Auch die Sicherheit in der Aufklärung von Patienten über die Unheilbarkeit der Erkrankung nahm nach dem PJ signifikant ab (p=0.0117), obgleich die Vorbereitung durch Q13 positiv hervorgehoben wurde.

**Schlussfolgerung: **Die im Q13 erworbenen Kenntnisse konnten im PJ nicht verankert werden. Im Gegenteil wurden die Gesprächsführung bei schwerer Erkrankung und der Umgang mit eigener Unsicherheit in der Behandlung von Schwerkranken von den Studierenden nach erster Praxiserfahrung als herausfordernd erachtet. Strukturelle Faktoren zu Palliativmedizin im PJ sowie intrinsische Motive der Studierenden, wie z.B. Vorwissen oder Lernmotivation, sollten analysiert werden, um Möglichkeiten zu erkennen, wie die Lücke zwischen theoretischer und praktischer Ausbildung in der allgemeinen Palliativversorgung geschlossen werden kann. Um ausreichend repräsentative Stichproben zu generieren, sollten zukünftige Studien zur Lehre für Studierende leicht zugänglich sein und attraktive Evaluationsformen unter Einbeziehung elektronischer Verfahren und Social Media berücksichtigen.

## Introduction

The introduction of palliative care into the medical curriculum in the 2013 summer term was an important step toward meeting the needs of around 400,000 people in Germany each year facing chronic terminal diseases or frailty at the end of life [1], [2], [3], [4]. Whenever providing medical care it is necessary to sufficiently consider the stresses associated with severe disease and death that are experienced by patients and their relatives on physical, psychological, social and existential levels. Alongside the development and expansion of structures to provide competent and dignified care to palliative patients and those at the end of life, a solid basic education in palliative care for all physicians with regard for existing staff resources is also important. For years now palliative care education has undergone continual development [5], [6], [7], [8], [9], [10]. The initial evaluation results of Q13 show improvement in palliative care education in the medical curriculum [11], [12], [13]. However, it remains open as to how effectively the acquired knowledge is consolidated in practice [9]. The consolidation of learning content in medical education is understood to be processes leading to long-term reproducibility of knowledge and skills [14].

**Figure 1:**
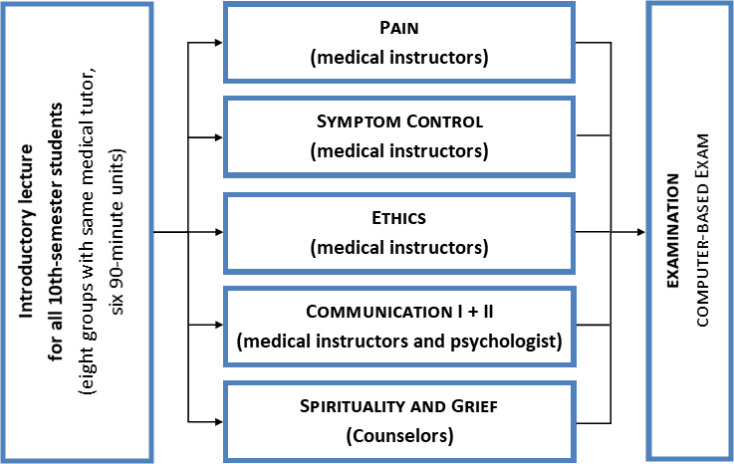
The multiprofessional course design for Q13 at the Medical School of the University of Mainz

The interdisciplinary subject palliative care (Q13), implemented in Mainz in the 2012 winter term, begins with an introductory lecture for all attendees and then goes on to cover five major topics in smaller groups (see figure 1 (Fig. 1)). The multiprofessional course design pursues the goal described in the literature of counteracting the inadequate confidence felt by students when interacting with the most severely ill [15]. All of the medical students in a semester were surveyed repeatedly to evaluate the course. The change in the students’ perceived level of confidence in different domains of palliative care (symptom control, psychosocial, ethical and spiritual aspects) was defined as the outcome and measured using questionnaires in the same cohort in a cross-sectional analysis before and after Q13 and again after the practical year (PY). The pre-post analysis before and immediately after Q13 has been previously published [11]. This study uses quantitative and qualitative methods to analyze the evaluation of Q13, attitudes and knowledge, self-confidence regarding palliative care and its related topics in students after Q13 and again in the same students after they had completed the PY.

## Materials und methods

### Study design

Descriptive prospective cohort study with a pre-post comparison of two data points (first immediately after Q13 and second after the PY) using quantitative (electronic) questionnaires and supplementary qualitative content-based analysis of the free-text comments. The influence of Q13 and the PY were analyzed for students’ sense of self-confidence in various dimensions of palliative care (anamnesis, content & basic knowledge, psychological aspects, pain medication, symptom control, communication, explaining change of therapeutic aim, explaining incurability, accompaniment of a dying patient, spiritual aspects).

#### Setting and sample

During the Q13 implementation phase at the University of Mainz, all of the tenth-semester students attending the compulsory course (N=176) were included. These students were asked to fill out a questionnaire prior to the start of Q13 and again at the conclusion of Q13 [11]. Over the course of the longitudinal analysis, this sample was surveyed a third time at the conclusion of the PY. This paper compares the data collected after Q13 and after the PY.

#### Measuring instruments

The questionnaire before and immediately after Q13 contains 17 questions to self-assess one’s own skills, evaluate Q13 in general, and the option to voice comments (see attachment 1 : questionnaire post-Q13 [15]). After the PY, students were additionally asked to what extent they found Q13 and/or the PY to be helpful in regard to the different categories (see attachment 2 : questionnaire post-PY). 

#### Data collection

The surveys were conducted during the introductory lecture (pre-Q13) and right after the written exam (post-Q13). The post-PY survey took place after completion of the PY but prior to the state medical examination. The pre-post questionnaires were filled out using pencil and paper during the Q13 course; the post-PY survey was electronic. Exactly one year after the post-Q13 survey the students were invited to participate in the post-PY survey via the e-learning platform ILIAS (Integriertes Lern-, Informations- und Arbeitskooperations-System) of the Center for Data Processing at the University of Mainz. The link to the online survey served not only to match that survey with the pre-post Q13 questionnaires, but also to explicitly anonymize the participants. At all three of the survey time points the students were asked to give a uniquely individual standardized code.

To reduce any potential selection bias toward particularly interested students with positive attitudes, the importance of critical feedback from the students was explicitly emphasized. In a bid to motivate as many students as possible to participation a third time in the Q13 survey during the learning phase, all of the participants were given access to final exam questions for the purpose of preparing for the state medical examination in return for their participation in the post-PY survey.

#### Statistical analysis of the quantitative data

The questions about self-confidence in regard to each of the Q13 topics are summarized by giving the absolute and relative frequencies for answers to single items. Scores for the somatic, psychological, spiritual, and knowledge dimensions, as well as total scores, were obtained from these 10 items. High values correspond with a high level of self-confidence. The scores are reported as the median and interquartile range (IQR). Comparisons between the survey time points are presented by giving the frequency of the changes (increases, decreases, no change) and the sign test.

The responses about how helpful Q13 and the PY were in regard to the topics in palliative care are also summarized using the absolute and relative frequencies for answers to single items. Comparisons between Q13 and the PY are made using Bowker’s test of symmetry [16].

In order to recognize any biases resulting from the different response rates for the three different survey time points, the participants’ answers were divided per survey. The participants in the survey after Q13 (post-Q13) were divided into those who had also participated in the survey after the PY (post-PY) and those who could not be matched with a post-PY questionnaire. The results of these groups were compared. Likewise, the participants of the post-PY survey were divided into two groups: those with matched questionnaires from the post-Q13 survey and those without. The frequency distributions were juxtaposed and compared using the Mann-Whitney test. The data analysis was carried out using SAS 9.4.

#### Content-based analysis of the qualitative data

The free-text comments submitted by the students after the 2011/12 winter term as part of the post-Q13 survey were qualitatively analyzed for content based on Mayring [17]. The main research questions were: How do students rate Q13? How confident do students feel dealing with topics in palliative care? The author (S.M.), a psychologist, and a doctoral candidate in medicine independently developed inductive categories that represent the core content of the statements. When assigning statements to the categories, consensus was found for statements that had been coded differently. This system of categorization was based on the free-text responses from the post-PY online survey and augmented to include categories appropriate to the research question about perceptions of palliative care during the PY and, in particular, the level of self-confidence when dealing with topics in palliative care. Following this, the categories for the first sample were coded by a third and independent research assistant (S.G., sociologist). Main categories were then developed from the inductively formed categories in the final step.

## Results

### Sample

Of the 176 students in the 2011/12 winter term, 165 (96%) filled out the questionnaire after Q13 and 79 (45%) after the PY. One dataset was excluded because the questions had been answered without actually completing the PY. Thirty-five questionnaires (20%) from the post-PY survey could be matched with post-Q13 questionnaires. The students whose questionnaires from the post-PY survey were matched with questionnaires from the post-Q13 survey did not differ statistically from those students whose questionnaires could not be matched.

#### Comparison of self-confidence post-Q13 and Post-PY

**Figure 2:**
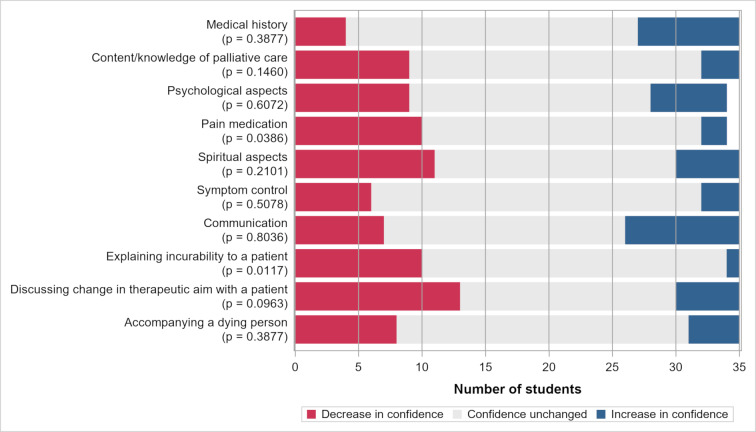
Changes in self-confidence when dealing with palliative care topics after the PY compared to after Q13. p-values from the sign test; horizontal bars are shortened in the case of missing values.

There were statistically noticeable changes in perceptions of confidence regarding drug-based pain therapy (p=0.0386) and explaining incurability to a patient (p=0.0117) (see figure 2 (Fig. 2)).

The vast majority of the 35 students whose responses could be matched to both surveys felt themselves to be (rather) confident immediately after Q13 and the PY in taking medical histories (80% post-Q13 and 91% post-PY), content and basic knowledge of palliative care (91% and 86%), pain medication (83% and 77%), symptom control (86% and 83%) and communication (77% for each respectively). In regard to psychological aspects, 74% and 60% felt themselves to be (rather) confident, 74% and 60% in terms of spiritual aspects, 54% and 40% about explaining the incurability of a disease to a patient, 63% and 54% about explaining changes in therapeutic aims to a patient, and 60% and 54% in accompanying the dying.

**Figure 3:**
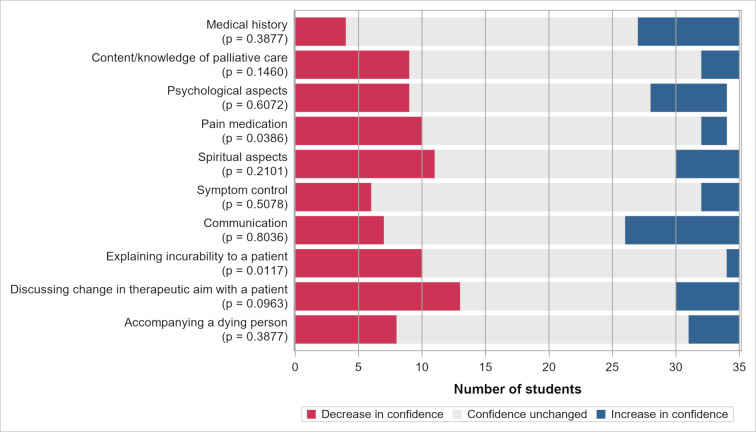
Changes in the summative data on one’s own knowledge of and interest in palliative care, self-confidence in interacting with the dying, and the assessment if working on a palliative care ward could be satisfying medically, after the PY compared to after Q13. p-values from the sign test; horizontal bars are shortened in the case of missing values.

For the summative items on a ten-point Likert scale, 49% of the students rated their knowledge of palliative care after the PY lower than directly after Q13; only 17% rated it higher (p=0.0347) (see figure 3 (Fig. 3)).

#### Subgroup analysis

There were hardly any differences regarding the learning content of the Q13 course in the statements on the post-Q13 survey by students whose questionnaires were matched to post-PY questionnaires and those for whom a match was not possible. Only for the topic of communication did students with paired post-PY questionnaires indicate a higher level of confidence (77% vs. 61%, p=0.0327). Immediately after Q13, these students reported more confidence in interacting with dying patients (p=0.0375) and claimed to be more interested in palliative care (p=0.0067) than after the PY.

#### Comparison of the benefit of Q13 and the PY

The students found the Q13 course to be definitively more educational than the internship year not only in terms of theory, but also practice. This affected not just topics pertaining to communication, psychology, and existential/spiritual aspects, which are actually only taught as part of palliative care, but also pain and symptom control (see table 1 (Tab. 1)).

#### Qualitative data

From 61 free-text comments on the post-Q13 survey in the 2011/12 winter term and 29 free-text comments after the PY, it was possible to categorize 122 and 78 statements, respectively.

In reference to the research question, the three categories, *general evaluation of the course – preparation for practice, self-confidence, und palliative care in the PY*, were identified during the qualitative content analysis as relevant main topics in the students’ free-text responses. It was possible to deductively apply and confirm inductively developed categories for the free-text analysis post-Q13 to the post-PY free-text responses. Table 2 (Tab. 2) presents an overview of the frequencies with which these main topics are mentioned by the students. The complete category system is presented in attachment 3 .

#### Evaluation of the course – preparation for practice

The *practical preparation* was especially highlighted in the course evaluation: “an excellently organized practicum, very practice-based” [post-Q13; student 10]. “In particular, Q13 helped me very much in conversing with patients and their family members” [post-PY; student 63]. The students explicitly mentioned the implementation of Q13 as making sense and rated the course as important: “I am glad that this topic has now finally found a way into medical study and has caught public attention. Death is part of life!” [post-PY; student 31]. The students’ statements generally support the result that they found the teaching in the Q13 course to be definitively more helpful than the PY in all regards. “Q13 helped me a lot in gathering experience with diagnostic and therapeutic measures to care for the dying or the terminally ill. I especially found the spiritual aspects and communication skills to be significant aids when interacting with such patients. I still see a need for improvement in my own development when making decisions in individual cases about discontinuing, or not starting, curative measures and when communicating diagnostic results” [post-PY; student 80].

#### Self-confidence

After Q13 one student described a persisting lack of confidence with the words: “One cannot feel certain prior to entering clinical practice and, above all, in interacting with the dying!”. Several participants also reported a lingering lack of confidence that they felt was normal after the PY. This feeling of uncertainty after the PY when explaining the incurability of a disease to a patient is illustrated by this example statement: “The role playing [in Q13] was good. I think that the course provided good preparation for patient interaction in palliative care during the PY. However, despite this, I repeatedly felt uncertain during the PY when confronted with real patients in real palliative situations. I think this is normal” [post-PY; student 72]. Uncertainty was perceived as “normal” during the PY. The first practical experiences come during the PY for which teaching in general, regardless of instruction on palliative care, cannot prepare a person: “It is difficult to evaluate if one feels generally confident in the care of a dying or most severely ill patient if one has never ever done it before! For this reason the teaching could have been as good as possible, which it was, and I still would have filled this questionnaire out the same as I have here. Ask me if I feel confident in treating a typical case of pneumonia: even here I must unfortunately respond in the negative because I may have an idea but I have not yet been able to put it into practice. Nevertheless, this subject has made a very important contribution to medical education...and should never be removed from the curriculum” [post-PY; student 74].

#### Palliative care in the PY

Much of the feedback after the PY refers explicitly to palliative care during the PY. Several students stated that they had no points of contact with palliative medicine during the PY: “Unfortunately, I did not have much contact with palliative care so that I was not able to sufficiently apply the knowledge that I had learned” [post-PY; student 15]. Despite the recognizable need for palliative care, the needs of patients are not responded to due to a lack of time: “Unfortunately this topic did receive anywhere near enough attention during the PY and it disturbed me how little patients were responded to for reasons of time” [post-PY; student 22]. Of course, positive experiences were also recounted: “During my practical year I experienced several patients in palliative situations. Some were very well cared for and it was interesting to see how palliative measures can very strongly influence the quality of life in a positive way during the final days” [post-PY; student 70].

## Discussion

The consolidation of learning content from the interdisciplinary subject palliative care during the practical year was investigated in a cohort of medical students. The comparison of the data collected after Q13 and after the PY showed that the majority of the students felt confident regarding palliative care. Exceptions to this are drug-based pain therapy and explaining the incurability of a disease. In terms of these two aspects students felt more uncertain after the PY than directly after Q13. This uncertainty was corroborated by an integrated qualitative analysis. Almost 50% of the participants felt that they had less knowledge of palliative care after the PY compared to the survey conducted after Q13; only 17% reported a subjective knowledge gain as a result of the PY. Overall, the Q13 course was evaluated by students as being more helpful for the development of their expertise and skills in palliative care than the PY.

This is the first study to longitudinally evaluate the compulsory palliative care education in Germany. There is an expectation in the international literature that as a result of including palliative care in the medical curriculum patient care will improve [18], [19], [20]. The consolidation of the knowledge taught using the teaching concept evaluated here has until now been little studied and focuses on structural features such as the teaching content [19], [21]. Direct proof of an improvement in palliative care for patients according to established evaluation models would be desirable. With this investigation of the level of self-confidence felt by future physicians it has been possible to focus in more closely on pertinent topics in palliative medicine [22], [23].

The strengths of this study stand in opposition to its limitations in that while a nonvalidated questionnaire was used, it had, however, already been proven in other studies [24], [25], and the validity of the survey results presented in this paper are supported by a supplementary qualitative analysis.

All the same, course evaluations as a way to ensure the quality of university teaching are disputed due to various risks of distortion [26]; because, for instance, the content analysis of the Q13 course in Mainz could have been positively influenced by the perceived dedication and motivation of the lecturers [27]. The remark of one student concerning recall bias [28] deserves attention given the survey time points in the longitudinal study: “I don’t know anymore how I responded a year ago” [post-PY; student 73]. Both of these biases skew in the same direction in reference to the difference in the evaluation of palliative care education assuming that the post-Q13 course evaluation is more positive than would be expected from the learning effect and that it involves a recall bias after the completion of the PY. Potential confounders and influencing variables of the effect include parameters of participant loyalty such as selection bias and non-response bias. Contradicting this is that the results of the paired questionnaires of students who participated post-Q13 (96% participation rate) and post- PY (45% participation rate) did not differ significantly from the students without matched questionnaires (participation traced only to one of the survey time points).

This study does not provide an answer to the question, whether or not the measured differences in perceived self-confidence are objectifiable or stem from mature perceptions of future physicians. However, the trend toward a decrease in perceived self-confidence and self-assessed knowledge across the entire cohort is striking.

### Generalizability

The phenomenon of decreasing self-assessed confidence and knowledge has been described previously [11], [29] and backs the assumption that the results for the participants reflect those for the total population. The higher level of self-confidence measured for communication skills in students with matched questionnaires suggests a bias more toward students with better social skills and the corresponding ability to engage in self-criticism. The Dunning-Kruger effect describes the tendency to overestimate one’s ability to apply new learning content, a tendency to which competent people are less susceptible [27]. This could, however, also involve a bias toward students who are more open to palliative medicine and for that reason lean toward giving more positive evaluations [30], meaning that it is possibly overestimated how much more helpful Q13 is compared to the PY.

In an analogous survey of students in Göttingen and Mainz prior to and after the PY, which in this case had been completed before implementation of Q13, substantial deficits were seen in perceived self-confidence and medical expertise regarding typical palliative care issues that had not improved during the PY [25]. In a US study, one year after taking the course only 45% of the students reported having good communication skills when it came to death and dying and advance directives, including DNR [31]. By contrast, the baseline level from which the cohort presented here started is higher, but even the generation of students with compulsory courses in palliative care had little opportunity during the PY to gather experience with treating palliative patients or to expand and deepen their knowledge.

What all of the studies share in common is a low participation rate – here 45%; in Weber et al. 29% [22]; in Parikh et al. 66% [31] – which, at least in part, can be linked to the difficultly in reaching students after they have graduated. Approaches to improve the participation rate could use social media or require participation in course evaluations.

## Conclusion

Our data show that future physicians are interested in topics pertaining to the care of the terminally ill and dying; however, the learned content from the interdisciplinary course in palliative care is seldom deepened during the practical year.

### Practical implications

The clear demand by students for more palliative care during the PY signalizes a need for action. Students value good teaching strategies and preparation for medical practice. This wish for more courses on holding conversations should certainly be satisfied in the practical preparation for the PY [11]]. It is precisely in providing care to terminally ill patients that uncertainties arise which cannot be sufficiently countered by merely strengthening theoretical knowledge in future physicians. Just as is the case in other disciplines, it is necessary to enable practical application of the knowledge learned, as intended during this phase of study. In view of demographic developments and medical advancements, it is therefore desirable that palliative care be given greater importance in the PY.

#### Research implications

The students in both groups (post-Q13/post-PY) did not differ at the different time points in their self-assessed self-confidence, but they did differ in the topics mentioned in the free-text comments. This finding supports the use of a mixed-methods approach in the evaluation of university teaching.

The perception of one’s own insecurity can be a mirror for an important learning objective in palliative care: working in a self-reflective manner, the effect of which on patient-centered communication and physicians’ satisfaction with their work could be a further topic for research. The change in self-confidence over the course of medical study and the possible difference in perception of uncertainty in newly qualified physicians following graduation from medical school in comparison to other disciplines are yet more research questions that arise from this study.

## Authors

Dr. Gerlach and Dipl.-Psych. Mai contributed equally to this work.

## Acknowledgements

We wish to thank all of the students for their valuable input on the Q13 evaluation and their participation in the survey, our colleagues who served as instructors, and Harald Affeldt (e-learning) from the Dept. of Research & Education at the Medical School of the University of Mainz for the technical support to create the e-survey and Clara Maßen and Swantje Goebel for their support in the qualitative analysis.

## Competing interests

CG received project funding for innovative teaching from the Gutenberg-Lehrkolleg (Gutenberg Teaching Council) at the Johannes Gutenberg University Mainz.

The other authors declare that they have no competing interests.

## Supplementary Material

Voluntary anonymous survey at the end of interdisciplinary subject Q13

Questionnaire PJ

Complete category system for the free-text comments submitted by the participating students (stud.) post-Q13 winter term 2011/12 and post-PY (N=90)

## Figures and Tables

**Table 1:**
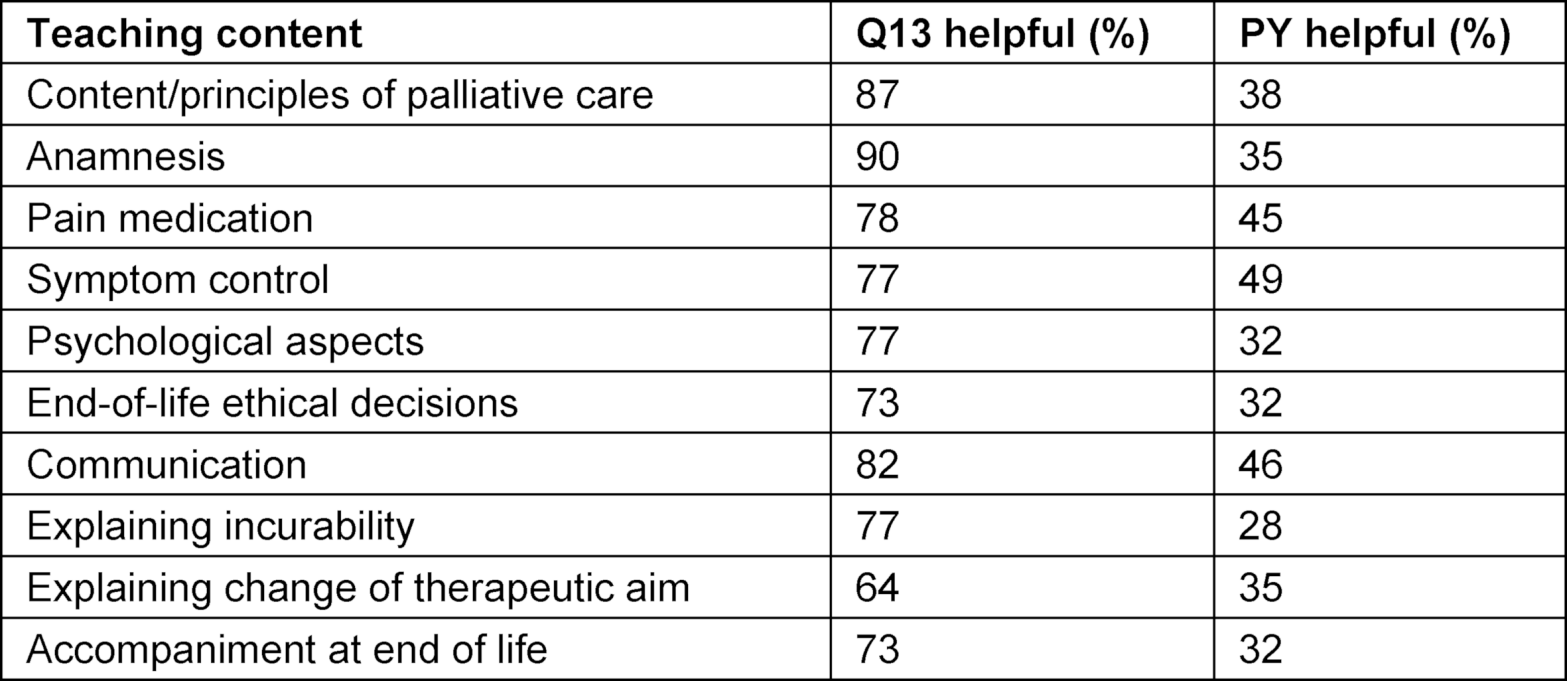
Student survey after the PY (N=78) on the benefit of the Q13 course and the PY in regard to palliative care topics; p<0.001

**Table 2:**
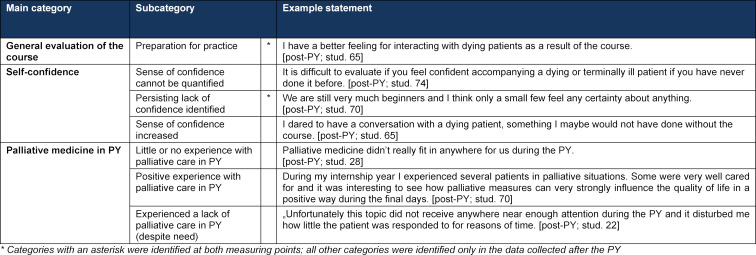
Categories for the free-text comments of the participating students (stud.) post-Q13 winter term 2011/12 and post-PY (N=90)
