# Supplementary material for: Palliative care in undergraduate medical education – consolidation of the learning contents of palliative care in the final academic year
Source: GMS J Med Educ. 2021 Sep 15;38(6):Doc103. doi: 10.3205/zma001499 (PMC8493850; doi:10.3205/zma001499)
Supplement: Voluntary anonymous survey at the end of interdisciplinary subject Q13 [file JME-38-6-103-s-001.pdf]

Attachment 1: Voluntary anonymous survey at the end of interdisciplinary subject Q13

In order to compare the results at the end of Q 13 anonymously and intra-individually with the results from the beginning of Q 13, please enter **your mother's birth date** in the following box just as you did for the first questionnaire.

(dd / mm / yyyy)

How do you rate your knowledge of palliative care (please circle the corresponding number)?

1	2	3	4	5	6	7	8	9	10

very low **very high**

How confident do you feel when interacting with severely ill and dying patients (please circle the corresponding number)?

1	2	3	4	5	6	7	8	9	10

very unconfident very confident

	**Confident**	**Rather confident**	**Rather unconfident**	**Unconfident**
When taking medical histories and examining patients with tumor pain, I feel:				
With the content and basic principles of palliative care, I feel:				
When integrating psychological aspects into the treatment and accompaniment of terminally ill and dying, I feel:				
When giving drug-based pain therapy to tumor patients, I feel:				
When integrating spiritual aspects into the treatment and accompaniment of the terminally ill and dying, I feel:				
When treating symptoms that can occur in the context of an advanced tumor disease, I feel:				
When communicating with the terminally ill and dying, I feel:				
When explaining the incurability of a patient's disease to the patient, I feel:				
When speaking with the patient about a switch from a causal/tumor-specific therapy (e.g. chemotherapy) to purely symptom-based therapy, I feel:				
When accompanying dying patients, I feel:				

I am interested in palliative care (please circle the corresponding number)

1	2	3	4	5	6	7	8	9	10

Does not apply at all Applies completely

I can imagine that working as a doctor on a palliative care ward can be satisfying (please circle the corresponding number).

1	2	3	4	5	6	7	8	9	10

Does not apply at all Applied completely

I find the implementation of the new interdisciplinary subject area palliative care makes sense (please circle the corresponding number).

1	2	3	4	5	6	7	8	9	10

Does not apply at all Applies completely

	Very good	Good	Less good	Not good
What is your overall evaluation of the teaching in interdisciplinary subject palliative care?				
How do you rate the script?				

What I would also like to say:
(Please write on the back as needed)

Thank You for filling out the questionnaire!

Attachment 1 to: Gerlach C, Mai SS, Schmidtmann I, Weber M. *Palliative care in undergraduate medical education – consolidation of the learning contents of palliative care in the final academic year.* GMS J Med Educ. 2021;38(6):Doc1499. DOI: 10.3205/zma00103
