# Supplementary material for: Palliative care in undergraduate medical education – consolidation of the learning contents of palliative care in the final academic year
Source: GMS J Med Educ. 2021 Sep 15;38(6):Doc103. doi: 10.3205/zma001499 (PMC8493850; doi:10.3205/zma001499)
Supplement: Questionnaire PJ [file JME-38-6-103-s-002.pdf]

Attachment 2: Questionnaire PJ

POST-PY Online Survey for Quality Assurance of the Teaching in Q13, Palliative Care. Addressee: Students attending the first interdisciplinary subject. Contact via email. Survey administered online, anonymized, data cannot be traced to the email addressee.

To enable a comparison of changes intra-individually and anonymously, please enter your mother's birth date as a code:

(dd / mm / yyyy)

Question 1

How would you rate your knowledge of palliative care today?

○	○	○	○	○	○	○	○	○	○	○
very low										very high

Question 2

How confident do you feel when interacting with terminally ill and dying patients?

○	○	○	○	○	○	○	○	○	○	○
very unsure										very sure

Question 3

I feel confident when taking medical histories and examining patients with tumor pain.

○	○	○	○
Disagree entirely	Mostly disagree	Mostly agree	Agree entirely

Question 3a

The teaching in the interdisciplinary subject palliative care has helped me in this respect.

○	○	○	○
Disagree entirely	Mostly disagree	Mostly agree	Agree entirely

Question 3b

I received helpful support <u>during the PY</u> when taking medical histories and examining patients with tumor pain.

○	○	○	○
Disagree entirely	Mostly disagree	Mostly agree	Agree entirely

Question 4
I feel confident regarding the content and basic knowledge of palliative care.<br>○ Disagree entirely ○ Mostly disagree ○ Mostly agree ○ Agree entirely
Question 4a
The teaching in the interdisciplinary subject palliative care has helped me in this respect.<br>○ Disagree entirely ○ Mostly disagree ○ Mostly agree ○ Agree entirely
Question 4b
I received helpful support with this during the PY.<br>○ Disagree entirely ○ Mostly disagree ○ Mostly agree ○ Agree entirely

Question 5
I feel confident integrating psychological aspects into the treatment and accompaniment of the terminally ill and dying.<br>○ Disagree entirely ○ Mostly disagree ○ Mostly agree ○ Agree entirely
Question 5a
The teaching in the interdisciplinary subject palliative care has helped me in this respect.<br>○ Disagree entirely ○ Mostly disagree ○ Mostly agree ○ Agree entirely
Question 5b
I received helpful support with this during the PY.<br>○ Disagree entirely ○ Mostly disagree ○ Mostly agree ○ Agree entirely

Question 6			
I feel confident giving drug-based pain therapy to tumor patients.			
○ Disagree entirely	○ Mostly disagree	○ Mostly agree	○ Agree entirely
Question 6a			
The teaching in the interdisciplinary subject palliative care has helped me in this respect.			
○ Disagree entirely	○ Mostly disagree	○ Mostly agree	○ Agree entirely
Question 6b			
I received helpful support with this <u>during the PY</u>.			
○ Disagree entirely	○ Mostly disagree	○ Mostly agree	○ Agree entirely

Question 7			
I feel confident integrating spiritual aspects into the treatment and accompaniment of the terminally ill and dying.			
○ Disagree entirely	○ Mostly disagree	○ Mostly agree	○ Agree entirely
Question 7a			
The teaching in the interdisciplinary subject palliative care has helped me in this respect.			
○ Disagree entirely	○ Mostly disagree	○ Mostly agree	○ Agree entirely
Question 7b			
I received helpful support with this <u>during the PY</u>.			
○ Disagree entirely	○ Mostly disagree	○ Mostly agree	○ Agree entirely

Question 8
I feel confident treating symptoms that can occur in the context of advanced tumor disease.<br><br>○ Disagree entirely ○ Mostly disagree ○ Mostly agree ○ Agree entirely
Question 8a
The teaching in the interdisciplinary subject palliative care has helped me in this respect.<br><br>○ Disagree entirely ○ Mostly disagree ○ Mostly agree ○ Agree entirely
Question 8b
I received helpful support with this during the PY.<br><br>○ Disagree entirely ○ Mostly disagree ○ Mostly agree ○ Agree entirely

Question 9
I feel confident communicating with the terminally ill and dying.<br><br>○ Disagree entirely ○ Mostly disagree ○ Mostly agree ○ Agree entirely
Question 9a
The teaching in the interdisciplinary subject palliative care has helped me in this respect.<br><br>○ Disagree entirely ○ Mostly disagree ○ Mostly agree ○ Agree entirely
Question 9b
I received helpful support with this during the PY.<br><br>○ Disagree entirely ○ Mostly disagree ○ Mostly agree ○ Agree entirely

Question 10
I feel confident explaining the incurability of a patient’s disease to the patient.
○ Disagree entirely ○ Mostly disagree ○ Mostly agree ○ Agree entirely
Question 10a
The teaching in the interdisciplinary subject palliative care has helped me in this respect.
○ Disagree entirely ○ Mostly disagree ○ Mostly agree ○ Agree entirely
Question 10b
I received helpful support with this during the PY.
○ Disagree entirely ○ Mostly disagree ○ Mostly agree ○ Agree entirely

Question 11
I feel confident speaking with the patient about a switch from a causal/tumor-specific therapy (e.g. chemotherapy) to purely symptom-based therapy.
○ Disagree entirely ○ Mostly disagree ○ Mostly agree ○ Agree entirely
Question 11a
The teaching in the interdisciplinary subject palliative care has helped me in this respect.
○ Disagree entirely ○ Mostly disagree ○ Mostly agree ○ Agree entirely
Question 11b
I received helpful support with this during the PY.
○ Disagree entirely ○ Mostly disagree ○ Mostly agree ○ Agree entirely

Question 12
I feel confident accompanying a dying patient.<br><br>○ Disagree entirely ○ Mostly disagree ○ Mostly agree ○ Agree entirely<br><br>Question 12a
The teaching in the interdisciplinary subject palliative care has helped me in this respect.<br><br>○ Disagree entirely ○ Mostly disagree ○ Mostly agree ○ Agree entirely<br><br>Question 12b
I received helpful support with this <u>during the PY</u>.<br><br>○ Disagree entirely ○ Mostly disagree ○ Mostly agree ○ Agree entirely

Question 13
I feel confident discussing ethical decisions at the end of life.<br><br>○ Disagree entirely ○ Mostly disagree ○ Mostly agree ○ Agree entirely<br><br>Question 13a
The teaching in the interdisciplinary subject palliative care has helped me in this respect.<br><br>○ Disagree entirely ○ Mostly disagree ○ Mostly agree ○ Agree entirely<br><br>Question 13b
I received helpful support with this <u>during the PY</u>.<br><br>○ Disagree entirely ○ Mostly disagree ○ Mostly agree ○ Agree entirely

Question 14
I am interested in palliative care.
○ ○ ○ ○ ○ ○ ○ ○ ○ ○ ○
Does not apply at all … Applies completely

Question 15
I can imagine that working as a doctor on a palliative care ward can be satisfying.
○ ○ ○ ○ ○ ○ ○ ○ ○ ○ ○
Does not apply at all … Applies completely

Question 16
The script for the interdisciplinary subject palliative care was helpful to me during the PY.
○ ○ ○ ○ ○ ○ ○ ○ ○ ○ ○
Does not apply at all … Applies completely

Question 17
The teaching in the interdisciplinary subject palliative care gave me confidence during the PY.
○ ○ ○ ○ ○ ○ ○ ○ ○ ○ ○
Does not apply at all … Applies completely

Free-text box for your comments<br>Response:
