# Supplementary material for: Palliative care in undergraduate medical education – consolidation of the learning contents of palliative care in the final academic year
Source: GMS J Med Educ. 2021 Sep 15;38(6):Doc103. doi: 10.3205/zma001499 (PMC8493850; doi:10.3205/zma001499)
Supplement: Complete category system for the free-text comments submitted by the participating students (stud.) post-Q13 winter term 2011/12 and post-PY (N=90) [file JME-38-6-103-s-003.pdf]

Attachment 3: Complete category system for the free-text comments submitted by the participating students (stud.) post-Q13 winter term 2011/12 and post-PY (N = 90)

Main category	Subcategory		Example statement
**General evaluation of the course**	Good course	*	Q13 was really good! [post-Q13; stud. 30]
	Important course	*	I consider this subject to be important and interesting [post-Q13; stud. 25]
	Implementation of course makes sense	*	Basically, I find that the implementation of this subject really makes sense. [post-Q13; stud. 35]
	Overall positive evaluation	*	Again, a lot of praise. [post-PY; stud. 51]
	Content overlap with other subjects	Q13	A lot overlapped with Medical Psychology & Sociology from the preclinical phase. [post-Q13; stud. 50]
	Good course/positive teaching	*	Well put together in terms of teaching and method [post-Q13; stud. 14]
	Preparation for practice	*	I have a better feeling for interacting with dying patients as a result of the course. [post-PY; stud. 65]
**Time point Q13**		*	Please, not in the 10th semester! [post-Q13; stud. 27]
**Evaluation of the organization for Q13**	Positive evaluation of the organization	*	One of the best organized subjected of the entire medical curriculum [post-Q13; stud. 1]
	Negative evaluation of the organization	Q13	The subject would suffice as a passive lecture course and avoid some of the resentment felt by student, or it should be offered to all students in the first half of the semester. [post-Q13; stud. 4]
	Group size, negative	Q13	Seminar group was too big. [post-Q13; stud. 6]
**Unit on communication**	Wish for more communication training	Q13	Above all, the communication trainings should be offered earlier in the curriculum and more often. [post-Q13; stud. 15]
	Positive emphasis on the conversations with simulated patients	*	The work with the actors was especially good! [post-PY; stud. 78]
	Overall negative evaluation	Q13	I had really hoped for tips on problem-solving in the sessions on communication. [post-Q13; stud. 50]
**Script**	Script positively rated	*	Script is super! [post-Q13; stud. 37]
	Script should be improved	Q13	Perhaps give more attention to medication. [post-Q13; stud. 3]
	Editing comments about the script	Q13	Script should be shorter → highlight the most important information. [post-Q13; stud. 9]

Attachment 3 to: Gerlach C, Mai SS, Schmidtmann I, Weber M. *Palliative care in undergraduate medical education – consolidation of the learning contents of palliative care in the final academic year.* GMS J Med Educ. 2021;38(6):Doc1499. DOI: 10.3205/zma00103

**Exam**	Difficult test	*	Test was hard! [post-Q13; stud. 5]
	Fair test	Q13	Test was fair & appropriate. [post-Q13; stud. 12]
	Test unnecessary	Q13	Test isn't necessary. [post-Q13; stud. 6]
**Evaluation of the instructors**	Positive mention of motivation	*	Super motivation on the part of the teachers! [post-Q13; stud. 32]
	Positive mention of the mentorship	Q13	Great guidance. [post-Q13; stud. 10]
	Gratitude	*	Thank you! [post-Q13; stud. 14]
**Criticism of the survey**	Criticism of method	PY	I don't know anymore what I decided on a year ago in TED. [post-PY; stud. 73]
**Self-confidence**	Sense of confidence cannot be quantified	PY	It is difficult to evaluate if you feel confident accompanying a dying or terminally ill patient if you have never done it before. [post-PY; stud. 74]
	Persisting lack of confidence identified	*	We are still very much beginners and I think only a small few feel any certainty about anything. [post-PY; stud. 70]
	Sense of confidence increased	PY	I dared to have a conversation with a dying patient, something I maybe would not have done without the course. [post-PY; stud. 65]
**Palliative medicine in PY**	Little or no experience with palliative care in PY	PY	Palliative medicine didn't really fit in anywhere for us during the PY. [post-PY; stud. 28]
	Positive experience with palliative care in PY	PY	During my internship year I experienced several patients in palliative situations. Some were very well cared for and it was interesting to see how palliative measures can very strongly influence the quality of life in a positive way during the final days. [post-PY; stud. 70]
	Experienced a lack of palliative care in PY (despite need)	PY	Unfortunately this topic did not receive anywhere near enough attention during the PY and it disturbed me how little the patient was responded to for reasons of time. [post-PY; stud. 22]

** Categories with asterisks were found at both measuring points; categories marked "Q13" were found only at the post-Q13 measuring point; categories marked "PY" were found only at the post-PY measuring point.*

Light blue highlighting indicates the categories relevant to the research questions asked by the study and which are presented in Table 2 of the paper.
